# The Technological, Economic, and Strength Aspects of High-Frequency Buried Arc Welding Using the GMAW Rapid HF Process

**DOI:** 10.3390/ma18071490

**Published:** 2025-03-26

**Authors:** Krzysztof Kudła, Krzysztof Makles, Józef Iwaszko

**Affiliations:** 1 Department of Technology and Automation, Faculty of Mechanical Engineering, Czestochowa University of Technology, 21 Armii Krajowej Ave., 42-200 Czestochowa, Poland; 2 Department of Materials Engineering, Faculty of Production Engineering and Materials Technology, Czestochowa University of Technology, 19 Armii Krajowej Ave., 42-200 Czestochowa, Poland; jozef.iwaszko@pcz.pl

**Keywords:** buried arc, high-frequency pulsed arc, deep penetration, butt weld, fillet weld, rapid process, load-carrying capacity of weld, strength of joints, welding filler metal consumption

## Abstract

One of the prospective methods of robotic welding with a consumable electrode in shield gas metal arc welding is the GMAW Rapid HF process (GRHF, HF-high frequency), in which welded joints with deep penetration welds are obtained thanks to the specially programmed welding characteristics of the arc. A pulsed frequency equalized to 5000 Hz was used to achieve consumable electrode arc stabilization and improve penetration. This work consists of two main sections, including the research and analysis of wire electrode melting and weld pool formation in the innovative GRHF process and its influences on joint strength and the economic advantages of welding. As a result of our research and strength tests, as well as an image analysis of phenomena occurring in the welding arc and weld pool, assumptions were developed about the use of the GRHF process, which is characterized by deep penetration welds without welding imperfections that reduce the quality of the welded joints and their strength. Welding conditions and parameters leading to welded joints characterized by high relative strength related to the weight of the used filler material were proposed. As a result of our research, it was found that the use of welding processes with deep penetration leads to material savings related to the reduced consumption of filler materials while maintaining the required high strength of welded joints. Savings of filler materials reaching 80% were achieved compared with hitherto used methods. At the same time, the maximum load-carrying capacity of welding joints was maintained.

## 1. Introduction

Welded constructions usually consist of joints made with the application of two kinds of welds: fillet welds, the total percentage of which of the total number of the constructions in question may reach as high as 90%, and also butt welds. The proportions of welds are determined by the low level of difficulty of making fillet welds, by the absence of the necessity of chamfering materials to be joined, by the lower required qualifications of welders, and also by the dominating proportion of the mutually perpendicular arrangements of sheets and sections for obtaining latticed, openwork, or crate-like shapes in a construction.

Owing to robotization, welding is conducted virtually exclusively in the Flat Position, or, alternatively, in the Horizontal Position, which, in turn, makes it possible to apply higher parameters of current and voltage, meaning that welds penetrate deeply. In this manner, the assets of welding result from the possibility of avoiding the chamfering of even the thick elements of angular joints, T-joints, and cruciform ones in order to obtain butt or butt–fillet welds, approved for use by virtue of the Eurocode 3 standard [1]. These possibilities are a result of the application of the new variants of the GMAW process within the scope of highly efficient welding without the rotation of the welding arc, and this means a deep penetration buried welding arc can be obtained, which is a method located between classic welding in the shield of shielding gases with the application of the GMAW Conventional CV (GCCV, CV—Constant Voltage) method and a hybrid Laser–GMAW or Plasma–GMAW. In order to obtain stable burning conditions for a buried welding arc, it is required to apply special-purpose welding devices, guaranteeing that the voltage of a welding arc is controlled [2], or, alternatively, devices with an adjustable power source dynamic response before and after a short–circuit [3]. A different method for the stabilization of a buried welding arc is replacing a direct current on the power circuit with a unidirectional modulated one, with a frequency between 200 and 600 Hz, to concentrate the welding arc and increase penetration [4].

**Figure 1:**
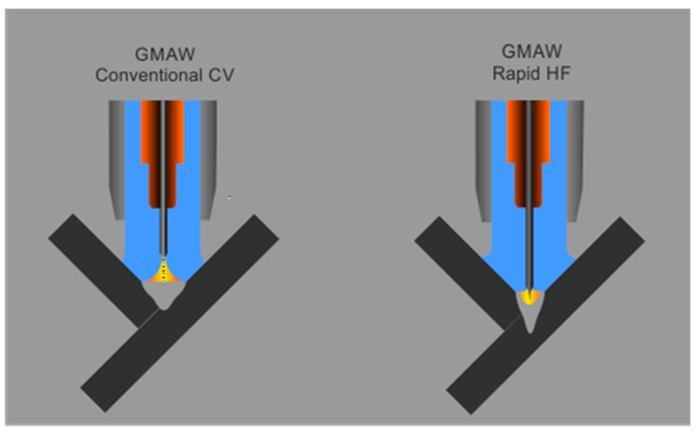
Penetrations of base metals in the GMAW Conventional CV and GMAW Rapid HF processes.

A solution that may become achievable in the future is the one presented in this work, consisting of the process of GMAW Rapid HF (Figure 1), in which a special-purpose programmed high-frequency source of electricity is applied in order to obtain the increasing power density of the heat source, bringing about deep penetration into a basic material. An important asset of the application of a welding arc of high frequency in comparison with different solutions is the significant reduction in the values of the welding current (to approximately 300 A) in cases where the welding arc is buried, and the transport of metal is stable. The application of high frequencies (exceeding 1000 Hz) decreases the sensitivity of the process to the disturbances of the work of a welding arc, in particular, in the case of the reduced value of the welding current without it being indispensable to apply an additional voltage control, preventing the unwelcome rotation of the welding arc. The frequency value (5000 Hz) was derived from the literature on the maximum pressure of the high-frequency arc (with an improved penetration capacity) and the metal transfer stability obtained in this study.

In similar solutions [5], in order to obtain a stable buried welding arc, higher values of welding current (approximately 600 A) are applied; alternatively, controlling that can also take advantage of a system based on additional voltage control [2,5]. The system proposed by Baba et al. [2], however, is based on welding within the scope of a comparatively low frequency of pulsation (approximately 100 Hz), which, in the case of welding currents within the scope of 400–500 A, brings about the porosity of welds, which results, among other factors, from disturbances in the transport of the metal to a welding pool.

The available literature does not provide detailed information related to the properties of joints made with the application of the GRHF process and the advantages that are possible to be achieved when taking advantage of a buried welding arc, bringing about obtaining deeply penetrated welds of high quality while simultaneously restricting the use of filler materials, bringing about a shortened arcing time, and, ipso facto, reduced electricity consumption.

**Figure 2:**
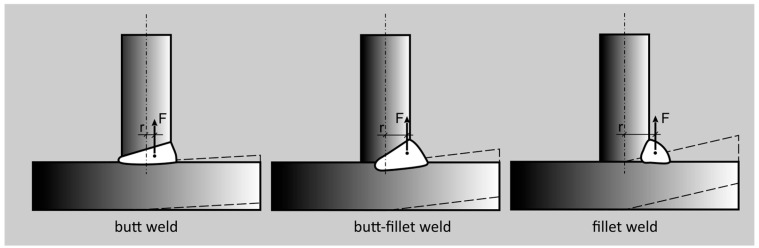
Illustration of the formation of angular distortion in T-joints connected with various types of welds (F-force causing joint deformation, r-arm of force F between the neutral axis and the center of gravity of the weld cross-section).

When applying deep penetration methods, it is possible to obtain (T-joint welds, cruciform welds, and different angular ones), instead of fillet welds, butt–fillet or butt ones, which decreases the volume and mass of construction, the consumption of filler metals, and also the size of angular welding distortions (owing to the advantageous relocation of the centers of gravity of welds in the area adjacent to the axis of the elements being joined), which is presented in Figure 2. As a result, it brings about constructions of higher classes.

**Figure 3:**
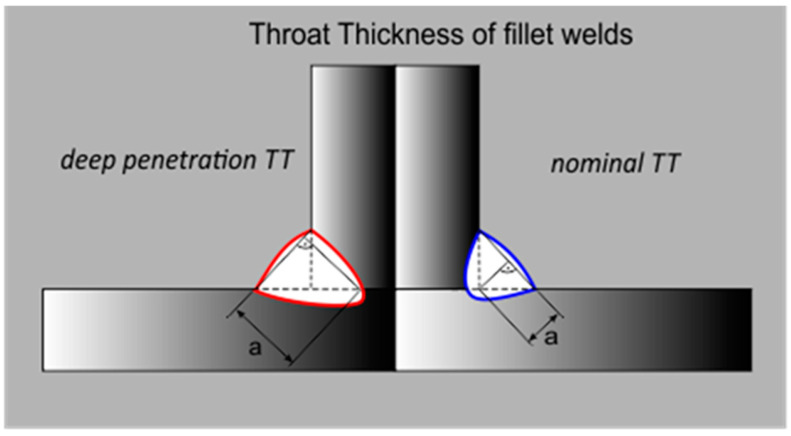
Throat thickness (TT) of fillet welds with and without deep penetration.

By using deep penetration methods of welding, it is possible to increase the effective throat of fillet welds (Figure 3), and, for this very reason, in accordance with the Eurocode 3 [1], while determining the calculated load-bearing cross-section capacity of fillet welds with deep material penetration, it is possible to take under consideration the additional throat of welds if the required depth of penetration may be regularly obtained (under the condition that this fact is documented). A similar approach to determining the effective throat of fillet welds with deep penetration is considered using different standards and legal regulations [6,7,8], which is presented in [9].

For several years, the most frequently made mistake has been that constructors commonly applied fillet welds with the following maximum values of throat: in the case of fillet welds made from both sides, a = 0.5t, or a = 0.7t_min_ in the case of one-sided ones. What is not taken into consideration is the depth of penetration on dimensioning fillet welds and treating them as if they were the butt–fillet or butt ones.

Making oneself acquainted with the solutions of contemporary welded constructions, one may conclude that the fillet welds applied to them are not designed (nor are they situated) with attention paid to individual needs, but instead designed and made without considering the actual requirements of construction and strength.

Nevertheless, the appropriate understanding of the impact of welds on construction may decrease their cost and reduce the final state of stresses and deformations, improving the properties in terms of the quality of workmanship, strength, and structural safety of the welded construction.

**Figure 4:**
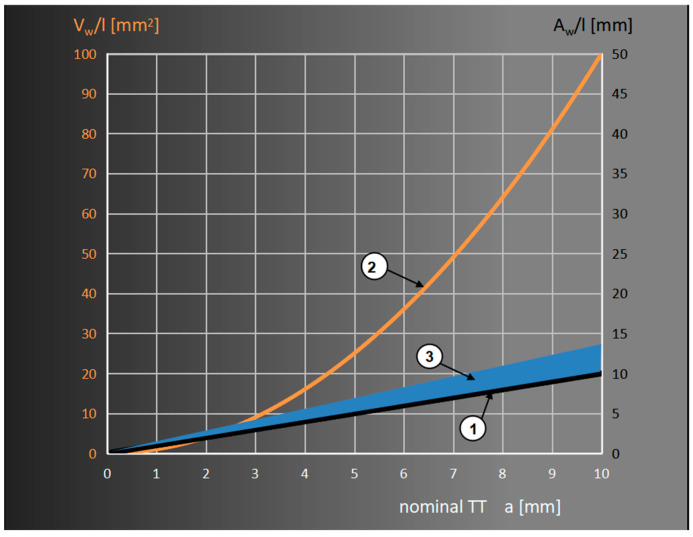
Difference of strength increase (1) and volume (2) of symmetrical fillet weld with flat surface. (3) The area of possible increase in strength due to the increased penetration allowed by the standard Eurocode 3 [10].

Because the volume of fillet welds increases in proportion to the square of its throat, whereas the strength is solely in proportion to the throat of fillet welds, and, therefore, increases much more slowly (Figure 4), the application of a large fillet weld size is not profitable, unlike inserting a weld inside the materials being joined, which achieves an increased effective throat, ipso facto the load-bearing cross-section of weld. For deeply penetrated welds, it does not excessively increase the volume and weight of construction, and the consumption of filler metals and the time of welding may be significantly reduced, whereas a simultaneous increase in strength is observed.

Following the assumptions presented in Figure 4, it is possible to observe that the volume of a fillet weld (V_w_), in comparison with its length (l), increases significantly more rapidly than its strength, whereas the load-carrying capacity of a butt weld is in proportion to the thickness of elements being joined.

It ought to be simultaneously ascertained that, in the case of the same throat of a fillet weld (a), the smallest field of cross-section, and, therefore, also its volume (respectively, A_w_ = a^2^ and V_w_ = a^2^l) are obtained for a flat fillet weld with equal legs. The larger the asymmetry of the welds and the larger the size, the more filler metal is used [11]. Similar losses are found to occur when the throat of fillet welds is excessive or when, while dimensions of a weld were being established, its actual penetration on a base material was not considered. This is accompanied by an increase in the unwanted deformations of a construction resulting from the formation of the concentration of stresses and the consequent development of cracks [12,13,14,15,16].

Modern welded constructions are designed with the application of critical states of load-bearing cross-section capacity [1], which means causing a more significant effort of crucial nodes and construction elements at a level approximately as high as their yield points. The stress concentration may bring about an excess in the load-bearing cross-section capacity, change in the static system of forces, and, in the further course, the initiation and development of cracks originating from the root and spreading toward the face of the welds [17]. The lack of joint penetration on the root of a weld may become a crucial point of the entire construction, particularly when it is exposed to the impact of changing stresses originated by external loads. For this very reason, a basic principle ought to be making deeply penetrated welds free of external geometrical notches and the lack of joint penetration exerting an influence on radical deterioration within the scope of mechanical properties, which is confirmed by numerous studies [18,19,20,21].

**Figure 5:**
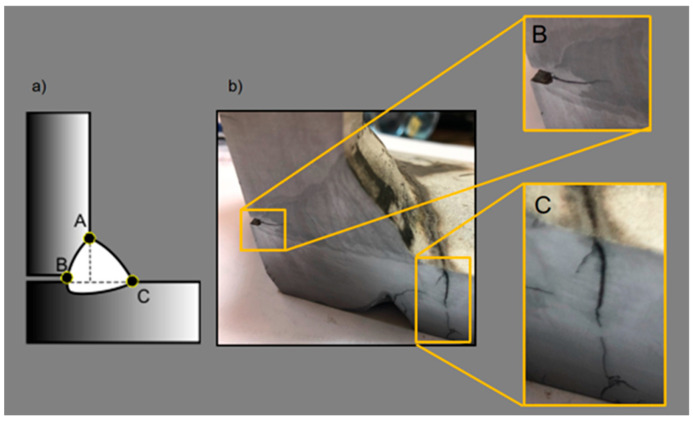
Cracking of T-joints: (**a**) critical points of a joint, (**b**) view of a joint after a long-term fatigue load.

The application of methods involving deep penetration may lead to the elimination of a critical part of those welds, especially the notches at the root (Figure 5), by achieving penetration on a previously made weld on welded joints on both sides or by making a one-side weld with complete joint penetration on both T-joints and cruciform ones.

## 2. Materials and Methods

In this work, a high-frequency pulsation current (5000 Hz) was applied to obtain a buried welding arc, bringing about a simultaneous shortening and concentration of a welding arc, increasing, ipso facto, the power density of the heat source. The advantage resulting from increasing the frequency of the pulsation of a welding arc is a simultaneous increase in pressure, which is exerted by it on a welding pool, bringing about its deepening and burying of a welding arc, bringing about the formation of a deep penetration and, simultaneously, avoiding the rotation of a welding arc—in turn, bringing about disturbances in the metal transfer and also undercuts.

**Figure 6:**
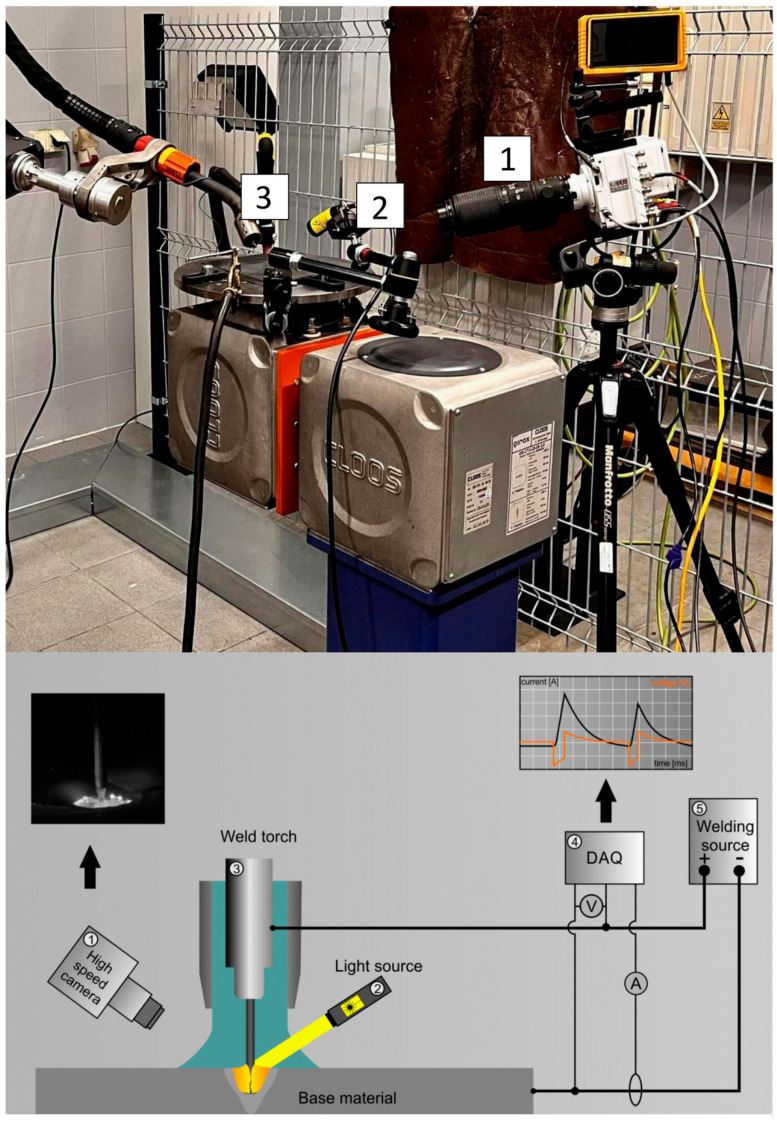
Scheme of the research station: 1—digital camera: PHOTRON 1024 PCI/PHANTOM VEO 710S, 2—light source: HMI lamp/laser Cavilux HF, 3—welding robot: Qirox QRC290, 4—measurement: DAQ NI USB-6251, 5—HF power welding source. The frequency of recording the image was 10,000 frames per second; the frequency of recording the parameters of current and voltage was 500,000 Hz.

To conduct the welding process, a synchronously connected high-frequency welding power Cloos Qineo Pulse 601 Pro (Carl Cloos Schweißtechnik GmbH, Haiger, Germany) and ESS Transtig 2704 (ESS Welding GmbH& Co. KG, Bad Waldsee, Germany), as well as a welding robot Cloos Qirox QRC 290 (Carl Cloos Schweißtechnik GmbH, Haiger, Germany), were applied. The power supply can achieve high-frequency pulsed current output with a frequency of up to 20 kHz. The recording of the time of current and voltage of a welding arc was made with the use NI USB-6251 (National Instruments, Austin, TX, USA), and 16 Channel DAQ Board (Photron Limited, San Diego, CA, USA) was synchronized with imaging space between electrodes and welding pools; it was achieved with the application of the measurement system presented in Figure 6. The arc phenomenon and molten pool formation were observed with high-speed cameras: PHOTRON 1024 PCI (Photron USA, San Diego, CA, USA) and PHANTOM VEO 710S (Vision Research, Wayne, MI, USA) with Sigma DG Macro 105 macro lens (Sigma GmbH, Rodermark, Germany). The frequency of recording the image was 10,000 frames per second, and the frequency of recording the current and voltage was 500,000 Hz.

The weld microstructure was observed by polishing the cut cross-section and then etching it with a 5% concentration of Nital solution for 5 s. The weld cross-sections were captured using Keyence VHX 7000 digital microscope (Keyence International, Osaka, Japan). VEB Fritz Heckert EU 100 tensile equipment (VEB FritzHeckert, Chemnitz, Germany) with maximum tensile force of 1000 kN) was used to test tensile strength according to ISO 9018:2016-01 [22].

The research was conducted for five kinds of steel: S355J2, S460NL, S700MC, S690QL, and 450HBW (Hardox), with thicknesses of, respectively, 8 + 8 mm (60 T joints TJ), 8 + 8 + 8 mm (60 cruciform joints CJ), 12 + 12 mm (60 TJ) 12 + 12 + 12 mm (60 CJ), 20 + 20 mm (60 TJ), and 20 + 20 + 20 mm (60 CJ). Filler metals were welding wires of the following kinds: G4Si1 and G69. The chemical composition of the welding wires and parent materials (mean value from Material Certificate for 8, 12, and 20 mm thickness base materials) is presented in Table 1. Welding conditions for joints shown in Section 3.3. are presented in Table 2.

## 3. Results and Discussion

### 3.1. Effect of the Impact of a High-Frequency Pulsation on the Weld Pool and Penetration on the Base Material

**Figure 7:**
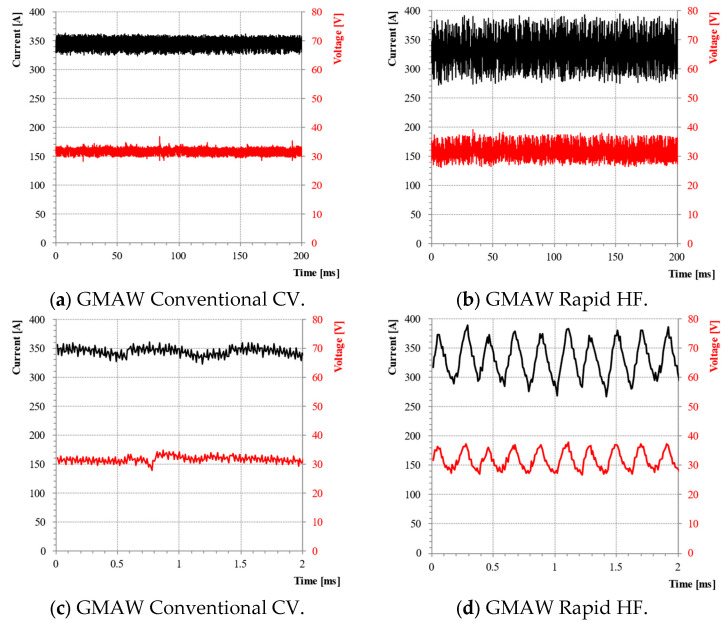
Welding current and voltage waveforms at wire speed welding of 11 m·min^−1^, non-pulsing arc, and the high-frequency pulsation of arc. (**a**,**b**) Typical waveforms during welding; (**c**,**d**) detailed graph of waveforms. 82% Ar 18% CO_2_, Ve = 11 m·min^−1^, CTWD = 16 mm.

**Figure 8:**
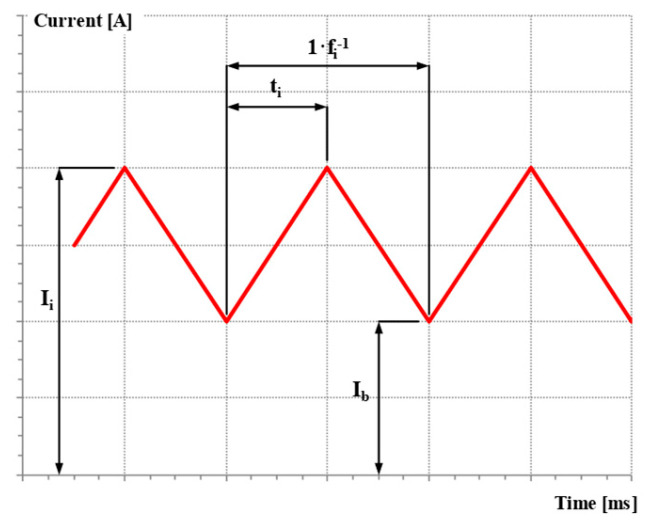
Schematic diagram of current waveform and pulse parameters for the GMAW Rapid HF process.

In Figure 7, the waveforms of current and voltage obtained using the GCCV process and the GRHF are presented. The parameters are collated in Table 3. The waveforms of the current and voltage for the GCCV process are characterized by a slight ripple (Figure 7c), resulting from the dynamic performance of power supplies and transistor used in controlled inverter, as well as the value of the inductance coil of the output filter, which restrics the ripples occurring in an output voltage and current waveforms. The obtained wave of pulse for the GRHF welding arc is the result obtained by the impulse mode of the welding source, which conducts programing and sets, for a given time, the flow of electricity determined by the following parameters (Figure 8): the pulse peak current I_i_, the pulse time t_i_, the frequency of pulsing f_i_, the base current I_b_, and the rising rates of the pulsed current dI·dt^−1^. The shape of the pulse is equivalent to a triangle wave.

The obtained characteristic waveforms of current and voltage results from the high frequency, in the case of which the time of the flow of the impulse amplitudes and base current is minimal. The principal role of the programmed high-frequency is stabilizing the burning of a buried welding arc and the axially controlled flow of liquid metal from the end of the electrode to the weld pool.

**Figure 9:**
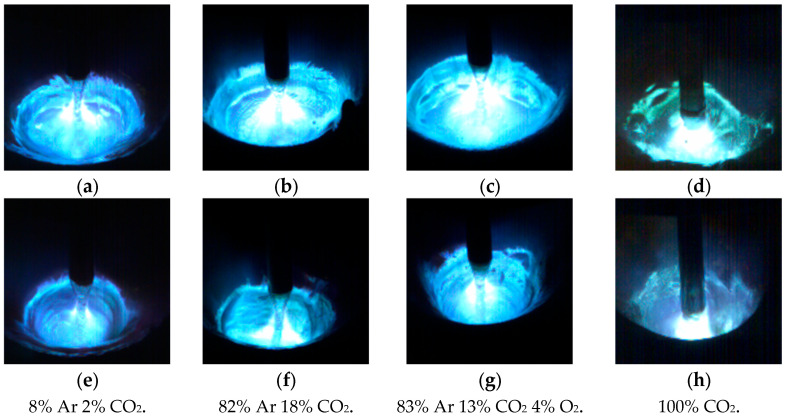
The comparison of typical welding arc profile taken using the high-speed video camera in studied types of welding arc: (**a**–**d**) GMAW Conventional CV; (**e**–**h**) GMAW Rapid HF welding arc. In the case of welding arc phenomena for pure CO_2_, the image was taken 1 millisecond after drop detachment from the wire tip, when the weld current value was about 330 A.

The value of a base current was assumed to be higher by approximately 40 A than the values of a critical current, in the case of which the form of the transport of metal is changed from globular to spraying for a typical shielding gas applied for welding structural steels of 82% Ar 18% CO_2_. Because the ionization of a welding arc and its concentration are strongly connected with the applied shielding gas, for that very reason, the scope of research was broadened by adding the analysis of the impact of the types of shield gas on forming a “keyhole” and the stabilization of a liquid transport of metal. In gases in which the principal ingredient is Ar, the applied impulse wave stabilizes the streaming transfer of metal. In the case of a gas shield of 100% CO_2_, the application of a pulsation of high frequency brings about a smaller visible dispersion of a metal vapor plasma under the wire tip. It also increases electrical conductivity and decreases the welding arc voltage, but the tapper formation leading to streaming transfer is not observed. Applying a high-frequency pulsation current with the value of 5000 Hz brings about a shortened welding arc, narrows the taper of the arc column, and creates a weld pool on a broad scope of the applied shielding gases, which is presented in Figure 9.

The value of the force with which a welding arc impacts a liquid pool is dependent on the square of the value of a current and also on the cross-section of the arc column, which is expressed by the following equation [26,27]:

(1) $$F_{\mathrm{arc}}=\frac{\mu_{0}\cdot I^{2}}{4\cdot \pi^{2}\cdot R^{2}}$$

where μ₀ is the magnetic permeability of the vacuum, I is the current, and R is the radius of a welding arc.

The principal expected effect of the application of high-frequency pulsation (exceeding 1000 Hz) will, therefore, be an indirect increase in the pressure on the welding arc [28], brought by about an increase in the temporary value of the current to approximately 380 A (the parameters of welding are shown in Table 3), as well as a decrease in the cross-section of an arc column and a direct increase in the depth of penetration in comparison with the GCCV process having the identical mean value of the welding current and voltage of the welding arc, which is also indicated by Krivtsun et al. [29]. In [28], it is shown how the pressure of the arc (and improvable penetration capacity) changes with the pulse frequency; up to about 5 kHz, it increases rapidly and above this value, it stabilizes at approximately four times the conventional DC value. Given these results, frequencies of 5 kHz were thought to be appropriate for welding application. The high frequency used in the tests determined the shortening of the pulse duration while the arc constriction minimized the current pulse time in the amplitude phase. The arc constriction was confirmed by phenomenology using high-speed photography as well as other works [30]. Periodic expansion and arc constriction are related to the current pulsation. If the duration of the maximum value (amplitude) is shortened, the cross-section of the arc column will also decrease at the peak current, leading to an increase in power density and, as a result, increased penetration. The increase in power density results from the current flowing through the arc column with a smaller cross-section.

**Figure 10:**
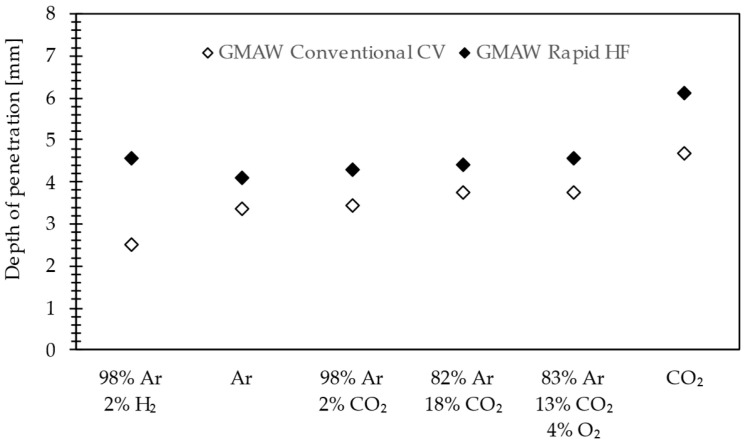
Depth of the penetration of GMAW Conventional CV and GMAW Rapid HF with different shielding gases.

**Figure 11:**
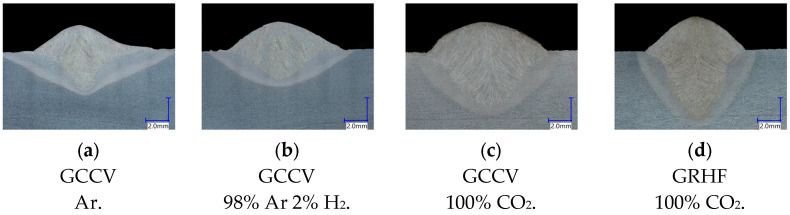
Some cross-sectional bead-on-plate weld tests presented in Figure 10.

**Figure 12:**
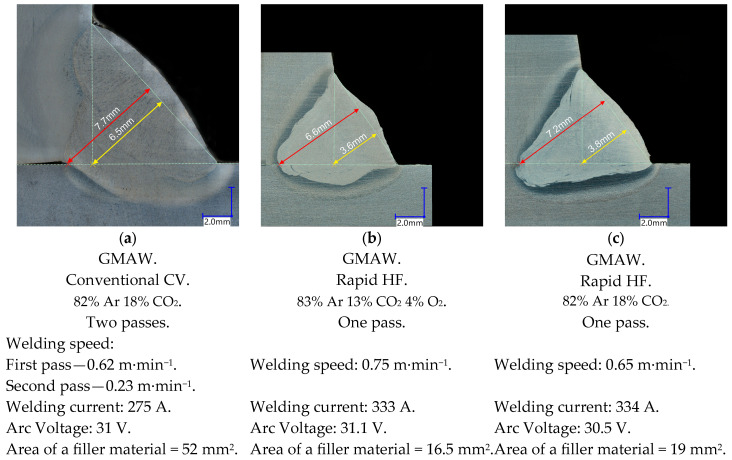
Comparison of fillet weld profiles with joints having equal throats. The red line determines the effective throat of fillet weld—a_eff_, and the yellow line indicates fillet weld size a.

The effect of the increasing penetration depth in the GRHF process can be observed regardless of the composition of the applied shielding gases. Figure 10 presents the effects of a high-frequency pulsed current compared with GCCV under different shielding gases. Beads on the plate were produced on unalloyed steel S355 J2 plates (200 × 200 × 10 mm) with the standard welding conditions of a wire speed rate of 11 m·min^−1^, welding speed of 75 cm·min^−1^, and CTWD (Contact Tip to Workpiece Distance) of 16 mm. The pulsed conditions are presented in Table 3. With the Ar-H_2_ shielding gas for GCCV the depth of penetration is smaller than pure argon because the thermal conductivity is significantly different for these gases. In the results of Ar-H_2_ shielding, the shape of penetration is bowl-shaped (Figure 11b) compared with the fingerlike penetration of the Ar-shielded welding arc (Figure 11a). The depth of penetration increases with an increase in the activated gas content. Welds produced under pure CO_2_ are relatively narrow and deeper, as presented in Figure 11c,d. These advantages are why this gas is widely used in the Japanese industry [31]. Increasing the CO_2_ (O_2_) content in the shielding gas makes penetration and fusion area larger, especially for content higher than 30% CO_2_. This phenomenon is attributed in the current literature [32] to a higher heat conductivity of CO_2_ compared with Ar (at high temperatures). Zielinska et al. [33] found that higher percentages of CO_2_ also produce wider arcs. A similar phenomenon is observed in the case of hydrogen addition [34]. Another explanation is that the increase in CO_2_ addition to the argon shielding gas changes the static characteristic of the welding arc, and resistance in the arc and arc energy rises. The essence of the application of the GRHF process is replacing fillet welds, the typical feature of which is small penetration and significant thickness (volume) by deeply penetrated welds (either fillet–butt or butt ones) made with the use of a significantly decreased quantity of the used welding filler metal. The analysis also included the influence of the composition of the shielding gas on the stability of the burning of a welding arc, and on producing a “keyhole”, and, consequently, on the penetration profile. In a broad scope of changes in the composition of shielding gases, welds having a deep penetration were obtained, which is presented in Figure 12 and confirms the effectiveness of applying the high-frequency GRHF process.

### 3.2. The Effect of the Impact of High-Frequency Pulsation on the Stabilization of a Buried Welding Arc

**Figure 13:**
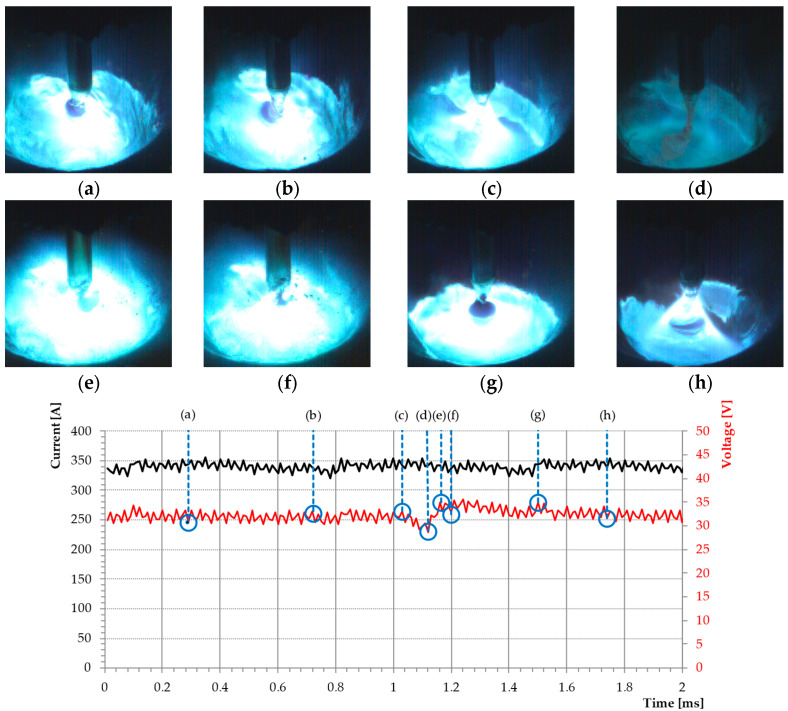
Arc phenomenon of the buried arc realized by CV power supply taken by synchronous high speed video camera (10 000 fps) and data acquisition card (500 000 Hz). 82% Ar 18% CO_2_, V_e_ = 11 m·min^−1^, CTWD = 16 mm.

Based on the conducted welding tests, the recorded waveforms of current and voltage, and imaging of the space between electrodes with the application of a high-speed video camera, it was ascertained that a high-frequency impulse current guarantees a significantly higher, in comparison with GCCV, stability of the process conducted with the application of a buried welding arc. In GCCV, we can observe a welding arc burning interruption brought about stochastically and found to occur, with short-circuits interrupting the stable streaming transfer, which is possible to be observed over time in the form of fluctuations in the voltage of a welding arc (point d on the graph in Figure 13). During short-circuits, we can observe a lack of stability in the metal flow from the electrode to the pool, bringing about the formation of welding spatter (Figure 13e). The observed instability of the transport of metal is found to occur in the lower ranges of a welding current (250–330 A) and the speed of the feed of a welding wire (9–11 m/min) matching a transition metal transfer, when, depending on the value of a voltage, projected transport is used instead (Figure 13g), and, in the further course, becomes a repelled one (Figure 13e), as a result of which a small droplet spatter is formed (Figure 13f). Similar disturbances were observed by Silva et al. [3], where, to stabilize the process, a change was made in the dynamic properties of the power source by regulating the speed of increasing current at the moment of a short-circuit.

**Figure 14:**
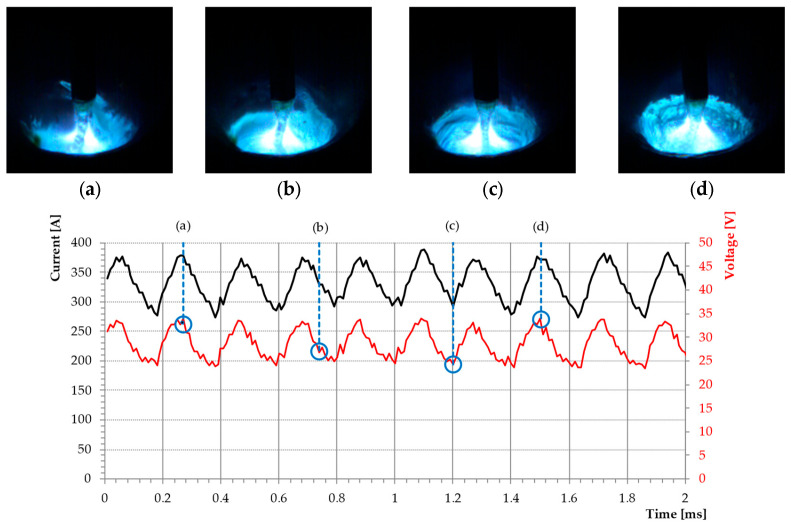
Welding arc phenomenon of the buried welding arc with the Rapid HF process taken by a synchronous high speed video camera (10 000 fps) and data acquisition card (500 000 Hz). 82% Ar 18% CO_2_, V_e_ = 11 m·min^−1^, CTWD = 16 mm.

Applying a high-frequency current on the innovative Rapid HF process in question brings about the concentrations of the arc column and increase of the power density while shortening the length of a welding arc, ipso facto, stabilizing the narrowing formed at the end of a welding wire. The obtained metal transport is characterized by homogeneous streaming transfer with minimal deviations of the stream of liquid metal from the wire axis, preventing an adverse rotation of a welding arc (Figure 14).

In the case of the application, as a gas shield, pure carbon dioxide, the melting of droplets assumes, similarly to the case of GCCV, a form of repelled transport. High-frequency guarantees, however, stabilizing the “keyhole” inside a welding pool, owing to which freely detached droplets do not leave a “keyhole” and do not form a spatter characteristic for welding with pure CO_2_ shielding gas. This fact opens new roads for the application. In the course of welding, carbon dioxide is a gas characterized by high thermal conductivity and constituting welds that have a favorable deep penetration while simultaneously minimizing the spatter. In the case of GCCV, it was observed that, in a liquid pool, a temporary disappearance of the “keyhole” occurs, and transport becomes irregular and repelled. Closing the “keyhole” is an unwanted effect because it does not guarantee obtaining a stable penetration, and increases spatters and forms different welding imperfections, which is also confirmed by Baba et al. [35].

### 3.3. The Assessment of the Effectiveness of Welding with the Application of the Rapid HF Process

To determine the influence of high-frequency buried welding arc, the GRHF technology on forming welds, and that of the use of filler metals consumption, research into 360 joints with fillet welds, fillet–butt and the butt ones, was performed on T-joints and cruciform welded (one- and both-sided). 

The joints were characterized by a changing nominal throat thickness of the welds. For further analyses, those that achieved the strength at the level of a parent material (PM) or maximum load-carrying capacity of weld were selected—the latter choice was made in the case of joints on which a deposited metal had a lower strength than PM and/or a rupture occurred on the area of welds. The results obtained were compared with identical joints made hitherto with the application of GCCV welding technology.

One of the objectives of conducting the above-mentioned research was to determine the possibility of decreasing the consumption of filler metals on welded joints while assuming a tendency toward the maximum load-carrying capacity of weld (a rupture on a base material or within the scope of the strength of base material).

To assess the effectiveness of the GRHF buried welding arc, the factor of the filler metals consumption expressed by Equation (2) were calculated. The assessment covered joints with a full or maximum load-carrying capacity of the weld made of base and filler materials collated in Table 1.

(2) $$A_{\mathrm{FM}}=\frac{\pi \cdot {d_{e}}^{2}}{4}\cdot \frac{V_{e}}{V_{s}}\cdot k$$

where d_e_ is the diameter of a welding wire (mm), V_e_ is the speed of the rate of a welding wire (m/min), vs. is the welding speed (m/min), and k is the number of welds (having those exact dimensions) on a joint.

**Figure 15:**
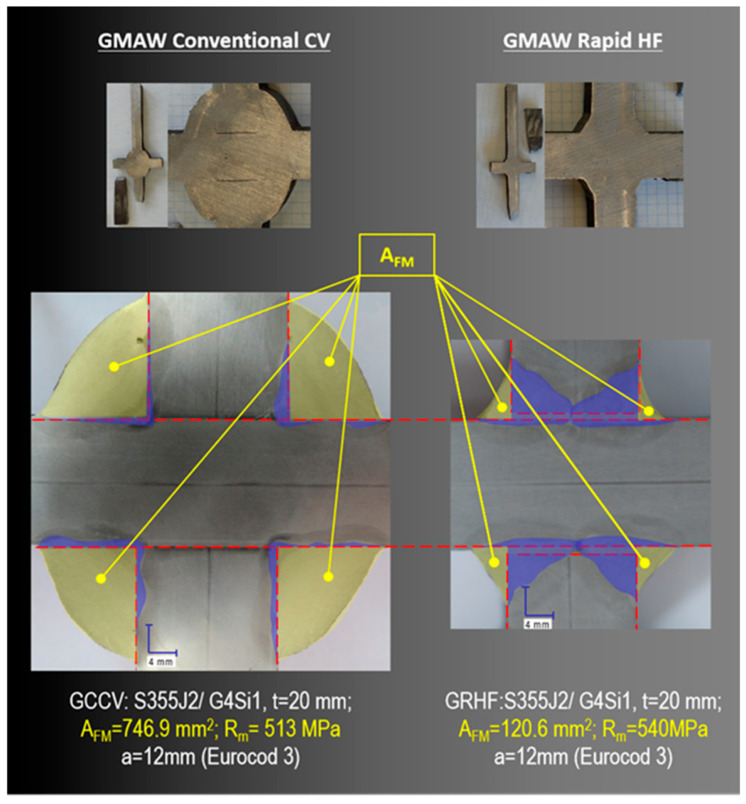
View of welded cruciform joints made with the application of the GMAW Conventional CV and GMAW Rapid HF processes (base material S355J2, filler wire G4Si1, and thickness t = 20/20/20 mm).

The factor of the filler metal consumption A_FM_ in mm^2^ presented in Equation (2) matches the cross-section of the parts of welds without the penetration area presented in Figure 15 (marked with the use of yellow color). Joints made with the application of the GCCV process are characteristic in the case of welds having low penetration (the penetration marked with the use of blue color) and also a substantial consumption of filler metals, whereas welded joints made with the application of the GRHF process have deeply penetrated welds made with a much smaller consumption of filler metals. What was obtained was the destruction (rupture) of welded joints at the level of strength of a base material, beyond areas where welds are situated. The obtained results prove it is justified to apply the GRHF process in restricting the consumption of filler metals while maintaining a high load-carrying capacity of joints with deep penetration welds.

**Figure 16:**
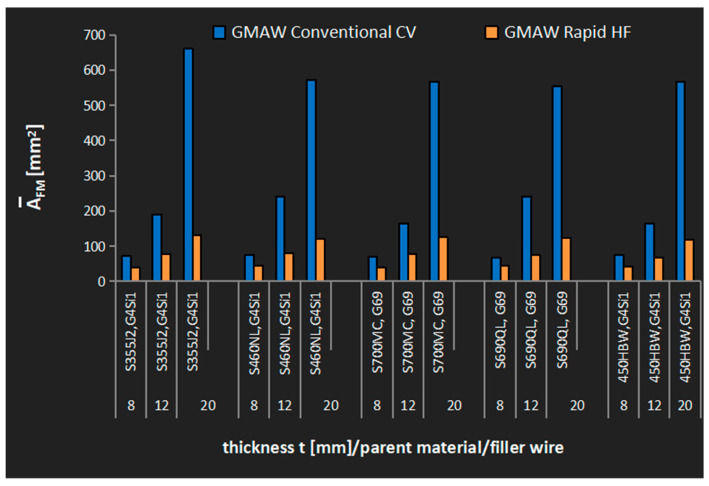
Mean value factor of the filler metal consumption $\bar{A}$_FM_ on cruciform welded joints with the application of the GMAW Conventional CV and GMAW Rapid HF processes.

Savings in the consumption of filler metals in the GRHF process compared with standard technology (GCCV) presented in Figure 16 fluctuated between 30–40% for joints with the thickness of t = 8 mm and 80% for joints with the thickness of t = 20 mm.

### 3.4. Analysis of the Load-Carrying Capacity of One-Side Fillet Welds/Fillet–Butt Welds Made with the Application of the GMAW Rapid HF Process

**Figure 17:**
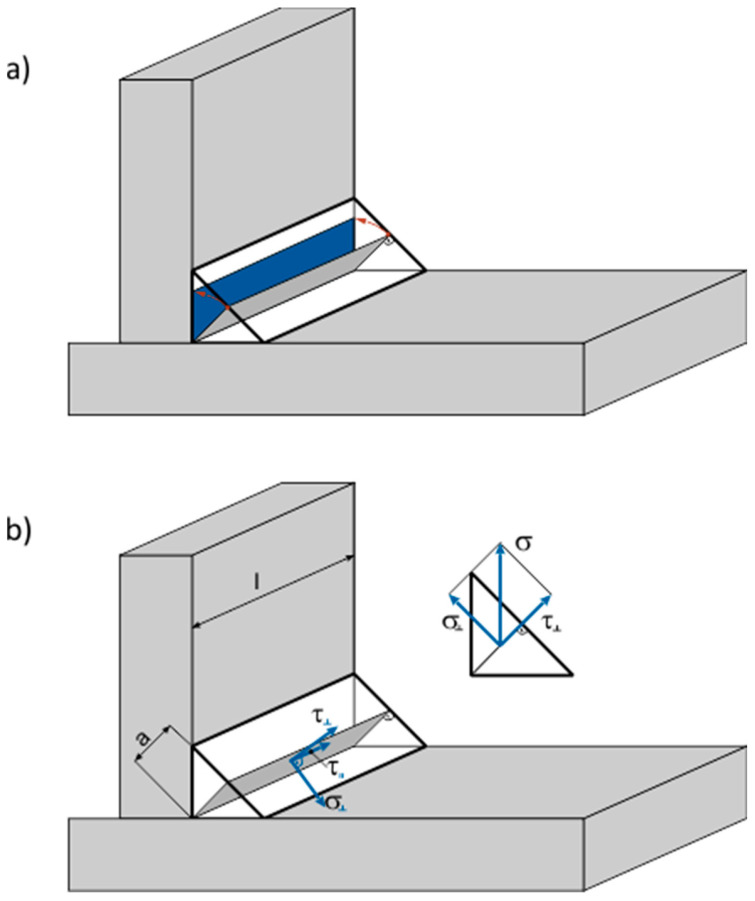
Operation of the section of a calculated root planes (bisector) of fillet welds on the plane of a drawing (**a**); distribution of external stresses into constituents—on the bisector plane of fillet welds (**b**).

Calculating the stresses originating from the external loads on fillet welds, conducted for 130 years according to the requirements of designing and making welded constructions, has undergone numerous changes and modifications. Initially, it was claimed that, as they were destroyed on a bisector plane, they ended up sharing [36]. It was assumed that by calculating all and any stresses, it is possible to determine the section of that plane at the angle of 45^0^ (for symmetrical welds) on the plane of the drawing (Figure 17a). The obtained field or section modulus/polar section modulus was seen as load-bearing factors while determining stresses, which always were of shearing character [37]. This method was not only paradoxical because it predicted tangent stresses occurring in three mutually perpendicular directions, but also, due to its lack of precision, it brought about a significant, even by several tens of percent, change in the dimensions of welds [38]. Its application is permitted by the Eurocode 3 [1], but as a simplified method because it does not require the distribution of forces impacting the bi-sectorial plane of welds, and, therefore, further calculations. Numerous studies in the field of the load-carrying capacity of fillet welds in the case of the complex states of stresses brought about, initially, as far as the specific cases were concerned, and in the further course more generally, determining the full ellipsoids of the strength of fillet welds for different steels [39], and, in the further course, determining appropriate models based on the hypothesis of Huber–Mises–Hencky [1,40]. In contrast to the simplified method, the directional method of the distribution of stresses on a bisector plane of welds is compatible with the actual mechanism of cracking fillet welds resulting from standard exploitation.

This method accurately represents the load-carrying capacity of fillet welds under the influence exerted by external loads. It is assumed that stress is not concentrated on the ends of welds, and the distribution of stresses is homogeneous all over the cross-section [40].

It ought to be indicated that, in the case of an angle different than that of 45 degrees, which means when its sine is not equal to cosine, calculations become somewhat more complicated because asymmetric fillet welds are applied. The lack of effectiveness of making and using fillet welds with unequal legs was proved in the authors’ own research [10]. The instance referred to hereinabove presents that the directional method of calculating fillet welds is quite simple and, however, brings about the practical and economical designing and application of fillet welds. The change in their dimensions (assuming, in the case of numerous constructions, an excessive leg size of welds) has been the main problem for many years in the case of welded constructions. Taking into consideration the fact that fillet welds outnumber butt ones and represent higher masses (to the degree reaching 9:1), it brings about excessive rigidity, fragility, and cracking of joints made even of steels with good weldability.

To compare the load-carrying capacity of welds made with the application of the GRHF process and GCCV, for assessment, T-joints made of the S355J2 steel with one-side welds were chosen, with the critical points of joints bringing about the formation of cracks and destruction from the root in the direction of weld faces, following the scheme presented in Figure 5.

**Figure 18:**
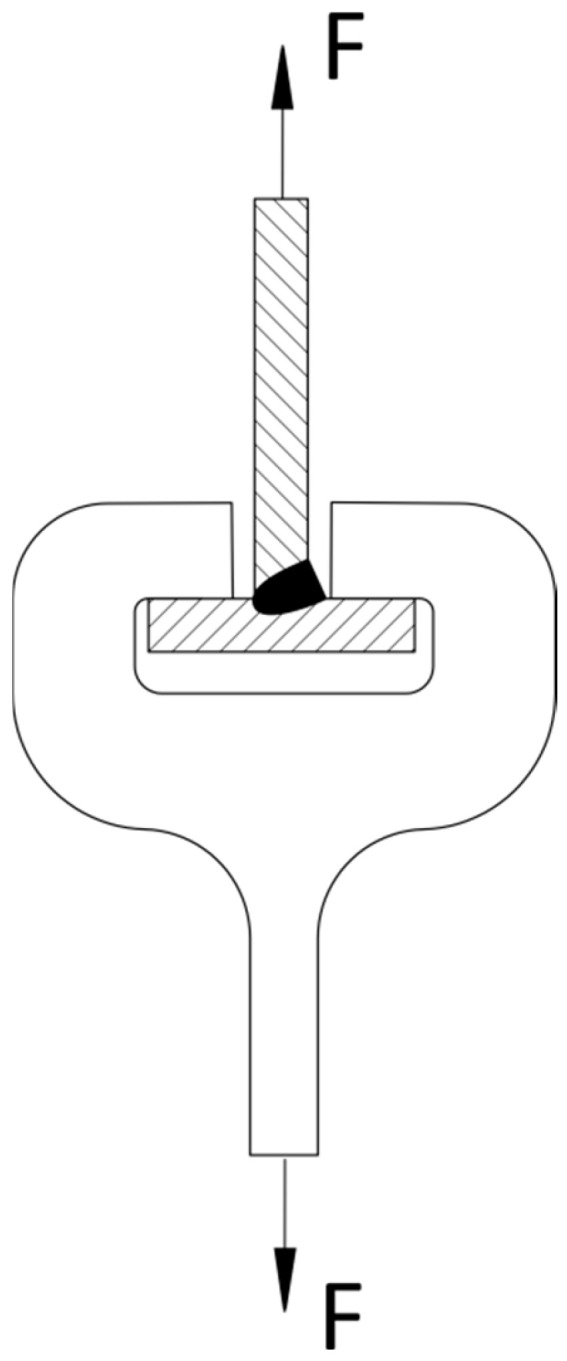
Direction and situation of the F force on the welded T-joints in the case of one-sided welds.

**Figure 19:**
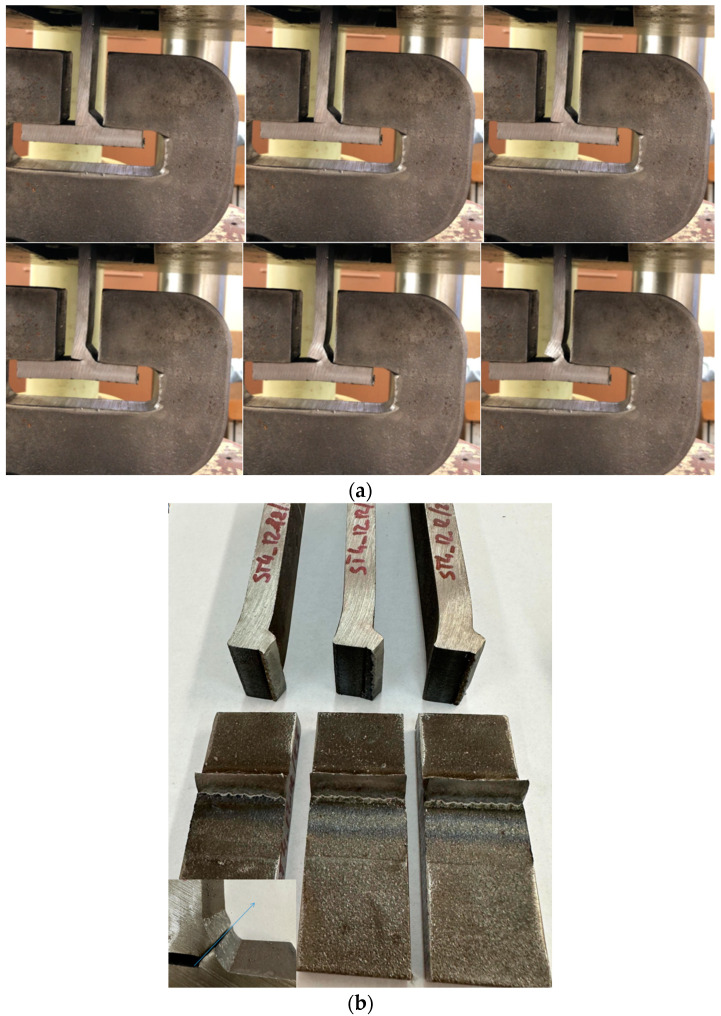
Destructive test of one-sided welds: (**a**) phases of destroying fillet/fillet–butt welds and (**b**) typical fractures of welded joints.

The calculation was conducted in accordance with the Eurocode. The following were assumed:

1. F force acts axially along the web of a T-joint (Figure 18);
2. The field of load-bearing cross-section welds in mm^2^ is as follows:
  (3) $$A_{w}=\;a\cdot l$$
  where a is the throat thickness calculated for fillet welds/fillet–butt welds (mm), and l is the length of fillet welds/fillet–butt welds (mm);
3. Welds (in accordance with the results of research) undergo destruction starting from the root point—on the axis of the joints in the direction of the weld face (Figure 19a);
4. Welds have the same transverse dimension on the entire length of welds.

Stress on fillet/fillet–butt welds amounts to the following:

(4) $$\sigma_{z}=\sqrt{\sigma_{⊥}^{2}+3\left(\tau_{⊥}^{2}+\tau_{∥}^{2}\right)\;}\leq \frac{f_{u}}{\beta_{w}\cdot \gamma_{M2}}$$

where σ_⊥_, τ_⊥_, and τ_∥_ are the constituents of the state of stresses on cross-section welds, respectively, normal and shear to the plane of cross-section; f_u_ is the nominal tensile strength of the weaker of the joined materials—within the scope of 360–560 MPa for t ≤ 40 mm.

(5) $$f_{u}=R_{m}$$

Β_w_ is the correlation co-efficient taking under consideration the higher mechanical properties of the material of a weld in comparison with the material (for the S355J2 steel Β_w_ = 0.85), and γ_M2_ = 1.25 is the factor of safety in the case of testing the load-bearing cross-section capacity on a rupture).

In the case of welds having a cross-section of symmetrical fillet welds,

(6) $$\tau_{⊥}=\frac{\sqrt{2}}{2}\frac{F}{A_{w}}=0.707\frac{F}{A_{w}}$$

Because shear stresses amount to τ_∥_ = 0 for the considered case, the equation of equivalent stresses on welds are as follows:

(7) $$\sigma_{z}=\sqrt{\sigma_{⊥}^{2}+3\tau_{⊥}^{2}}\leq \frac{f_{u}}{\beta_{w}\cdot \gamma_{M2}}$$

(8) $$\sigma_{z}=\frac{F\cdot \sqrt{2}}{A_{w}}$$

Thus calculated, equivalent stresses constitute a load-carrying capacity of welds in the case of the studied welded joints.

**Figure 20:**
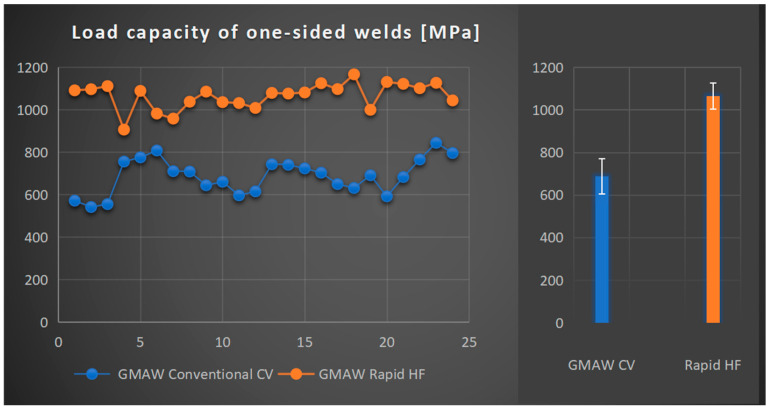
Load-bearing cross-section capacity of welds on joints made with the application of the GMAW Conventional CV method and the GMAW Rapid HF process (base material—S355J2, filler wire—G4Si1, shielding gas—82%Ar + 18%CO_2_).

In Figure 20, the results of destructive research on joints with one-side welds made with the application of the GCCV and GRHF are presented.

Conducted research into load-carrying capacity welds on the researched joints indicated its increase for a deeply penetrating GRHF welding in comparison with the one made with the hitherto in use GCCV. In particular, it is significant that there is an increase in the load-carrying capacity, which achieved the mean value of 1066 MPa (standard deviation 62 MPa), which, in comparison with GCCV welding of 688 MPa (standard deviation 83 MPa), constitutes an increase in the load-carrying capacity at the level of 55%, confirming, ipso facto, a decisive influence of deeper penetration on the load-carrying capacity of welds accompanied by a simultaneous decrease in the filler material consumption.

**Figure 21:**
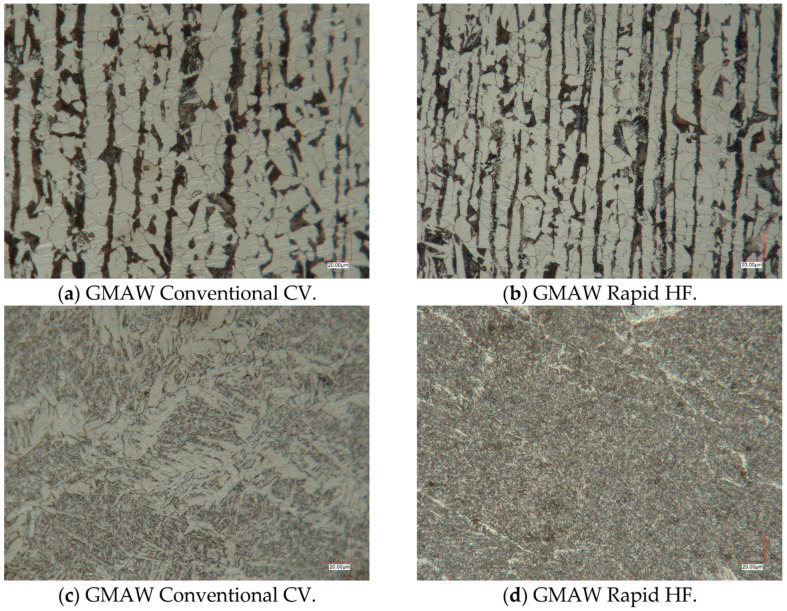
Exemplary microstructures in GCCV and GRHF joints: (**a**,**b**) base material; (**c**,**d**) central region of the welds (filler wire—G4Si1, shielding gas—82%Ar +18%CO_2_).

Micrographs of the base material (S355J2) and the welds of GCCV (first pass: welding current: 275 A, arc voltage: 31 V, welding speed: 0.62 m/min; second pass: welding current: 275 A, arc voltage: 31 V, welding speed: 0.23 m/min), and GRHF (welding current: 334 A, arc voltage: 30.5 V, welding speed: 0.65 m/min) welding were observed and compared. Figure 21 shows cross-sectional photographs of the central part of the welds made with both welding processes. In GCCV welding, the primary microstructures are coarse grain boundary ferrite and side plates ferrite, with small amounts of acicular ferrite. In contrast, weld metals produced with GRHF contain fine-grained boundary ferrite and a higher proportion of acicular ferrite.

The phenomenon of joint destruction is similar in both welding processes GCCV and GRHF. The initial cracks start at point B (Figure 5 in the article), located in a weld root, and then develop across a bisector plane of welds. The fracture is plastic (Figure 19b). The range of load carrying capacity was different, as shown on Figure 20. The greater load-bearing capacity of welds made with GRHF, compared to those made with GCCV, is primarily due to the greater effective weld throat and the higher content of acicular ferrite.

## 4. Conclusions

When applying the methods of deep penetration welding, including the GMAW Rapid HF process, it is possible to accomplish a few important objectives in construction and economical use. The joints are durable, and their creation requires relatively little energy consumption, whereas the carbon footprint is significantly decreased, and so is the consumption of filler metals; consequently, the time required to build the construction may be significantly shortened. These advantages are ever more significant in the case of an increase in the thickness of joined elements. The findings were confirmed by numerous tests conducted with the application of various types of constructional steels.

The innovative process of GMAW Rapid HF, based on a high-frequency impulse current, has a favorable influence on the stabilization of buried welding arc and also creates an effective increase in penetration on a base material in a broad scope of applied shielding gases. This technology may be successfully applied in the production of deeply penetrated fillet welds, characterized by an increasingly effective cross-section load-carrying capacity, while considering increased depth penetration calculated per the Eurocode 3 [1].

## Figures and Tables

**Table 1:** Chemical composition of welding wires and base materials (in wt-%).

Element	G4Si ^1^	G69 ^2^	S355J2	S460NL	S700MC	S690QL	450HBW
C	0.071	0.08	0.130	0.176	0.066	0.141	0.200
Si	0.91	0.60	0.018	0.455	0.034	0.288	0.350
Mn	1.63	1.70	1.49	1.68	1.90	1.10	1.32
P	0.070	-	0.150	0.011	0.008	0.010	0.015
S	0.014	-	0.050	0.0009	0.0005	0.0003	0.001
Cr	0.021	0.25	0.089	0.035	0.028	0.330	0.980
Ni	0.033	1.5	0.046	0.014	0.011	0.190	0.040
Al	0.002	-	0.068	0.020	0.054	0.057	0.087
Cu	0.106	-	0.052	0.011	0.011	0.295	0.020
Nb	-	-	0.025	0.002	0.047	0.023	0.030
Ti	0.009(Zr + Ti)	-	0.001	0.002	0.143	0.021	0.010
V	0.001	-	0.002	0.109	0.008	0.005	0.010
B	-	-	0.0000	0.0002	0.0002	0.0026	0.0030
Mo	0.013	0.3	0.010	0.011	0.004	0.167	0.170
N	-	-	0.006	0.025	0.000	0.0048	0.0400

^1^ ISO 14341:2020 [23]: G4Si1; DIN 8559: SG3; AWS A-5.18: ER 70S-6:S355J2, S460NL, 450HBW. ^2^ ISO 16834:2025 [24]: G69.4M Mn3Ni1CrMo; AWS A-5.28: ER 100 S-1: S700MC, S690QL.

**Table 2:** Welding parameters for the GCCV and the GRHF process with maximum cruciform joint strength.

Process	Wire Feed Rate [m·min^−1^]	Welding Speed [m·min^−1^]	CTWD [mm]	Pulse Frequency [Hz]	Thickness PM [mm]	Welding Current [A]	Arc Voltage [V]	Heat Input [kJ·mm^−1^]
GCCV	8.2	0.50	15	-	8	240	27.4	0.63
GCCV	8.0	0.15	15	-	12	250	20.0	1.60
	8.8 (1P)	0.60	15	-		254	30.9	0.63
GCCV	8.8 (2P)	0.15	15	-	20	252	31.0	2.50
	8.0 (3P)	0.15	15	-		235	22.5	1.69
GRHF	16	0.90	14	5000	8	430	34.0	0.78
GRHF	16	0.54	16	5000	12	430	36.0	1.38
GRHF	16	0.55	16	5000	20	430	39.0	1.46

Heat input calculated by ISO/TR 17671-1:2002 [25] (thermal efficiency ratio—0.8), CTWD—Contact Tip to Workpiece Distance, wire diameter—1.2 mm, shielding gas—M21, gas flow—15 l·min^−1^, (1P, 2P, and 3P)—first, second, and third pass in the weld.

**Table 3:** Welding conditions and detailed pulse parameters for the GCCV and the GRHF test presented in Figure 7.

Process	Wire Feed Rate [m·min^−1^]	Pulse Frequency [Hz]	Peak Current [A]	Pulse Time [ms]	Base Current [A]	Response Rate [A·ms^−1^]	Welding Current [A]	Arc Voltage [V]
GCCV	11	-	-	-	-	-	340	31.5
GRHF	11	5000	380	0.1	280	1000	330	28.5

## Data Availability

The original contributions presented in this study are included in the article. Further inquiries can be directed to the corresponding authors.
